# Evaluation of a multicomponent intervention to shorten thrombolytic door-to-needle time in stroke patients in China (MISSION): A cluster-randomized controlled trial

**DOI:** 10.1371/journal.pmed.1004034

**Published:** 2022-07-05

**Authors:** Wansi Zhong, Longting Lin, Xiaoxian Gong, Zhicai Chen, Yi Chen, Shenqiang Yan, Ying Zhou, Xuting Zhang, Haitao Hu, Lusha Tong, Chaochan Cheng, Qun Gu, Yong Chen, Xiaojin Yu, Yuhui Huang, Changzheng Yuan, Min Lou, for the MISSION investigators

**Affiliations:** 1 Department of Neurology, the Second Affiliated Hospital of Zhejiang University, School of Medicine, Hangzhou, China; 2 Imaging Laboratory Manger, Sydney Brain Center, University of New South Wales, Australia; 3 Department of Neurology, Yongkang First People’s Hospital, Yongkang, China; 4 Department of Neurology, Huzhou First People’s Hospital, Huzhou, China; 5 Department of Neurology, Li Huili Hospital of Ningbo Medical Center, Ningbo, China; 6 Key Laboratory of Environmental Medicine Engineering, Ministry of Education, School of Public Health, Southeast University, Nanjing, China; 7 Department of Big Data in Health Science, School of Public Health, Zhejiang University, Hangzhou, China; Columbia University, UNITED STATES

## Abstract

**Background:**

Rapid intravenous thrombolysis (IVT) for acute ischemic stroke (AIS) is crucial for improving outcomes. However, few randomized trials of interventions aimed at reducing in-hospital delay have been carried out in China. We aimed to evaluate the effect of a multicomponent intervention on thrombolytic door-to-needle time (DNT) of AIS patients via video teleconference based on the Behavior Change Wheel (BCW) method.

**Methods and findings:**

This cluster-randomized trial, conducted between January 1, 2019 and December 31, 2019, randomly allocated 22 hospitals equally to PEITEM (Persuasion Environment reconstruction Incentivization Training Education Modeling) intervention or routine care plus stroke registry and subsequently enrolled 1,634 AIS patients receiving IVT within 4.5 hours upon stroke onset from participant hospitals. The PEITEM group received a 1-year PEITEM 6-component intervention based on the behavioral theory monthly via video teleconference. The primary outcome was the proportion of patients with a DNT of 60 minutes or less. A total of 987 patients participated in the PEITEM group (mean age, 69 years; female, 411 [41.6%]) and 647 patients in the control group (mean age, 70 years; female, 238 [36.8%]). Of all participants, the proportion of DNT ≤60 minutes in the PEITEM group was higher than in the control group (82.0% versus 73.3%; adjusted odds ratio, 1.77; 95% confidence interval (CI), 1.17 to 2.70; ICC, 0.04; *P* = 0.007). Among secondary outcomes, the average DNT was 43 minutes in the PEITEM group and 50 minutes in the control group (adjusted mean difference: −8.83; 95% CI, −14.03 to −3.64; ICC, 0.12; *P* = 0.001). Favorable functional outcome (score of 0 to 1 on the modified Rankin scale (mRS)) was achieved in 55.6% patients of the PEITEM group and 50.4% of the control group (adjusted odds ratio, 1.38; 95% CI, 1.00 to 1.90; ICC, 0.01; *P =* 0.049). Main study limitations include non-blinding of clinicians, and that specific interventions component responsible for the observed changes could not be determined.

**Conclusions:**

The teleconference-delivered PEITEM intervention resulted in a moderate but clinically relevant shorter DNT and better functional outcome in AIS patients receiving IVT.

**Trial registration:**

Clinicaltrials.gov NCT03317639.

## Abstract 2

Wansi Zhong and co-workers study an intervention aimed at improving delivery of prompt thrombolytic treatment for ischemic stroke in China.

## Author summary

**Why was this study done?:**

- An earlier administration of intravenous thrombolysis (IVT) is associated with a lower risk of in-hospital mortality and better functional outcome in acute ischemic stroke (AIS) patients. Even a small improvement in door-to-needle time (DNT) could have significant benefit to patient outcomes in China.
- The improvement of DNT is of great urgency in China as Chinese patients were reported twice as likely as non-Chinese patients to have DNT >60 minutes.
- Applying behavioral therapy to intervention improvement, such as the Behavior Change Wheel (BCW) method, has been successfully used in multiple disciplines.

**What did the researchers do and find?:**

- We conducted a cluster-randomized controlled trial to explore whether a multicomponent behavior change intervention on hospital personnel via video teleconference could increase the proportion of DNT ≤60 minutes in patients receiving IVT in China.
- We enrolled 1,634 patients from 22 hospitals. Hospitals were allocated equally to receive PEITEM (Persuasion Environment reconstruction Incentivization Training Education Modeling) intervention based on BCW method or routine care plus stroke registry.
- The intervention group had a higher proportion of patients with DNT ≤60 minutes (82.0%) than the control group (73.7%) and had more cases with no or minor neurologic deficits at 90 days after stroke onset.

**What do these findings mean?:**

- The teleconference-delivered multicomponent behavior intervention was associated with a shorter DNT and higher rate of favorable functional outcome in AIS patients in China.
- This study indicated the successful application of behavioral therapy in the improvement of stroke care quality, which could be further implemented in the current global Coronavirus Disease 2019 (COVID-19) pandemic crisis.
- Though these comprehensive interventions were feasible and effective at the cluster level, it is not sure whether individual intervention would have the same effect.

## Introduction

The benefit of intravenous thrombolysis (IVT) improving the prognosis of acute ischemic stroke (AIS) patients is time-dependent [1–4]. An earlier administration of IVT is associated with a lower risk of in-hospital mortality and hemorrhagic transformation and better functional outcomes at 90 days after onset [5–7]. The American Heart Association/American Stroke Association (AHA/ASA) guidelines thus recommend a door-to-needle time (DNT) less than 60 minutes for IVT [1] The AHA/ASA Target: Stroke Initiative also has a goal to have 75% of all patients treated within 60 minutes of hospital arrival [8].

China faces the greatest challenge from stroke in the world, with an estimated death rate of 149.49 per 100,000 in 2018 [9]. However, the reported median DNT in 2011 was 116 minutes in China, and only 17.8% of IVT achieved DNT less than 60 minutes [10]. Moreover, Chinese patients were reported to be twice as likely as non-Chinese patients to have DNT more than 60 minutes [11]. While public hospitals, accounting for 92% of hospital admissions in China, are overcrowded with patients, and clinicians in most stroke centers have limited time and resources to receive complex medical intervention, a comprehensive program is clearly demanded in China, with ongoing monitoring of DNT as an important benchmark for quality in stroke care.

As reported in previous studies, DNT could be reduced via multiple strategies such as moving patients to computed tomography (CT) on emergency medical service stretcher, starting alteplase treatment in CT suite, prenotification of an incoming stroke patient and not waiting for blood test unless indicated [12–15]. The practical implementation of these interventions requires refinement of workflow and passion of clinicians in stroke quality improvement. Recently, a pre-post cohort study in Alberta has demonstrated that an improvement collaborative implementing multifaceted behavioral interventions among clinicians was likely the key contributing factor in reducing DNT and improving outcomes for AIS [16]. However, behavioral interventions to shorten DNT still needed to be validated in randomized clinical trials.

The Behavior Change Wheel (BCW) is a new approach applying behavioral therapy to intervention improvement [17]. It has been used to improve medication management in multimorbidity, smoking cessation care, medication adherence, and inpatient stroke rehabilitation [18–21]. A combination of a theoretically driven, comprehensive intervention strategy may have the potential to make a major contribution to shorten DNT. With the development of internet technology, teleconference is increasingly applied in medical training to clinicians to improve the intervention. Therefore, we conducted a cluster-randomized clinical trial to explore whether a multicomponent behavior change intervention on hospital personnel based on BCW method via video teleconference could increase the proportion of DNT ≤60 minutes in AIS receiving IVT in China.

## Methods

### Study design

This clinical trial, entitled as Improving In-hospital Stroke Service Utilization (MISSION), was an open-label, multicenter cluster-randomized controlled trial. We divided study activities into 2 periods: preintervention period (January 2018 to December 2018) and intervention period (January 2019 to December 2019). The study is reported according to the CONSORT guidance for reporting cluster-randomized trial (**S1 Checklist**) [22]. The trial protocol was described in detail in **S1 Text**. The human ethics committee of the Second Affiliated Hospital of Zhejiang University (SAHZU), School of Medicine, approved the trial protocol. The clinical trial was conducted according to the principle expressed in the Declaration of Helsinki. Written informed consents were obtained from the participating hospital staff. Informed consents were also obtained from patients or their legally authorized representatives for treatment with alteplase and participation in a telephone survey in accordance with the local hospital standards.

### Study participants

Hospitals in the Zhejiang Stroke Alliance meeting the inclusion criteria were eligible for the study: (1) agree to participate in ongoing data report and continuous audit of IVT processes of care and outcomes; (2) have a stroke unit or staffing equivalent to a stroke physician and a nurse. Nevertheless, hospitals with fewer than 20 thrombolytic cases per year were excluded. Only AIS patients receiving IVT within 4.5 hours upon stroke onset were included.

### Randomization and masking

We randomly stratified the hospitals according to their baseline proportion of IVT patients with DNT ≤60 minutes (<60%, ≥60% to ≤72%, >72%) collected in preintervention period (January 2018 to December 2018). These clusters were randomized 1:1 to a multicomponent intervention via video teleconference (PEITEM (Persuasion Environment reconstruction Incentivization Training Education Modeling) group) or routine care and stroke registry participation (control group) by using a computer-generated randomization sequence. Masking of study and hospital personnel to site assignment was not possible because of the nature of the intervention. However, external clinical evaluators assigned to assess the outcomes were masked.

### Data collection

Data on all thrombolytic patients were consecutively recorded in a secure, purpose-built web-based data entry system, which was only accessible to those approved to login. A local trained study-specific delegate, who was independent from the study team, entered patient data at each participating hospital. The trial management group monitored and checked the quality of the data submitted by the participating hospitals.

### Intervention

Hospitals assigned to the PEITEM group implemented the PEITEM intervention from January 1, 2019 through December 31, 2019 based on the behavioral theory using the BCW method [17]. The BCW emphasizes the importance of ensuring that proponents have the capability, opportunity, and motivation to perform the desired behavior. The PEITEM intervention, consisted of 6 major components including **P**ersuasion, **E**nvironmental reconstruction, **I**ncentivization, **T**raining, **E**ducation and **M**odeling, was implemented to stroke team and emergency department team by a professional medical quality care initiative (QCI) team. IVT was delivered by the neurologists in stroke team.

Persuasion and Modeling aimed to foster communication between clinicians and research team to stimulate clinician’s action to shorten DNT and to provide a good example to follow. A series of measures were embedded such as setting up DNT target, time tracking with feedback, case discussion, and championship experience presentation to decrease in-hospital delay. Training and Education could help to improve the skills and knowledge of the clinicians. The Education involved the early identification of eligible patients for IVT, rapid decision-making based on the rapid risk evaluation of hemorrhagic transformation and complication management, and the training of sustainable application of quality improvement tools; the purpose was to help rapid diagnosis, decrease initial refusal, and improve complication management. Incentivization encouraged stroke doctors to create expectation of reward. We established a pioneer award according to the number of IVT patients and the proportion of DNT ≤60 minutes. Finally, we implemented Environmental reconstruction as a persist intervention by inserting the standardized medical record templates involving evidence-based performance measures in the electronic medical record system of all hospitals in the intervention group, aiming to change the physical context of patients’ information. The team members had to attend a 2-hour video tele-conference monthly. A summary of the PEITEM intervention and its link with BCW is shown (**Fig A in S2 Text)** and a detailed description is given in **Table A in S2 Text**.

### Outcomes

The primary outcome was the proportion of DNT ≤60 minutes in AIS patients treated with IVT, indicating fast thrombolytic treatment upon hospital arrival. The secondary outcomes included the key time intervals between assessment and thrombolytic treatment (DNT, onset-to-needle time (ONT)), modified Rankin scale (mRS) score and death at discharge, complication after IVT (symptomatic intracranial hemorrhage (sICH)), and favorable functional outcome at 90 days (score of 0 to 1 on the mRS), indicating the efficacy and safety of the PEITEM intervention. DNT referred to the time between hospital arrival and initiation of IVT. ONT was the time between symptom onset and IVT initiation. sICH was defined as intracranial hemorrhage at 24 hours associated with an increase of ≥4 points of National Institutes of Health Stroke Scale (NIHSS) score from baseline, according to European Cooperative Acute Stroke Study (ECASS) II trial [23].

### Sample size

A prerandomization survey at participating clusters was conducted. In addition, based on the outcomes reported by previous studies, the sample size estimation considered that a change from 55% to 70% of patients with DNT ≤60 minutes would be clinically important [24]. Therefore, a total of 1,424 patients from 22 hospitals would be required to detect a 15% improvement in AIS patients with DNT ≤60 minutes, with 80% power, 5% significance level, and an intracluster correlation coefficient (ICC) of 0.05.

### Data analysis

We used the intention-to-treat analysis for all outcomes. We presented the baseline characteristics of hospitals and patients through summarizing the continuous variables as median with interquartile ranges and categorical variables as frequency and percentage. We performed Wilcoxon rank-sum test and Chi-square test to compare the continuous variables and categorical variables, respectively. Modes were used to impute missing values of categorical variables. Only prior antiplatelet usage had missing data at 3.1%.

ICC was calculated using the correlation-based estimation methods for categorical outcomes and analysis of variance methods for continuous variables, respectively [25]. Generalized estimating equation models with an exchangeable working correlation structure were conducted to account for the correlations of observations within clusters (hospitals) [26]. Odds ratios for the binary outcomes (DNT ≤60 minutes, sICH, favorable functional outcome at 90 days, and death at discharge) and mean differences for the continuous outcomes (DNT, ONT, and mRS score at discharge) were estimated to assess the treatment effects. Multivariable model for DNT ≤60 minutes, DNT, and ONT adjusted for patient characteristics (including age, sex, history of stroke/TIA, hypertension, diabetes, atrial fibrillation, coronary heart disease, prior antiplatelet usage, thrombectomy, smoking, prestroke mRS score, and NIHSS score at admission) and hospital characteristics (including hospital grade (tertiary and secondary), stroke unit, teaching hospital status, and annual stroke discharge). Clinical outcomes adjusted for age, prestroke mRS score, NIHSS score at admission, and other patient characteristics with a *P* value of <0.1 in the univariate analysis.

Additionally, to assess whether the improvement in primary and secondary outcomes in the PEITEM group was influenced by the time window, we also conducted a sensitivity analysis with the additional inclusion of patients receiving IVT beyond 4.5 hours upon stroke onset. All patients receiving IVT beyond 4.5 hours upon stroke onset had CT or MRI core/perfusion mismatch. For patients lost to follow-up at 90 days, we used multiple imputation (5 times) as a sensitivity analysis and compared the baseline characteristics between patients with and without mRS at 90 days. A 2-tailed *P* < 0.05 was considered statistically significant. All analyses were performed using SAS (version 9.4) and R software (version 4.0.5).

## Results

### Hospital and patients’ characteristics

**Fig 1:**
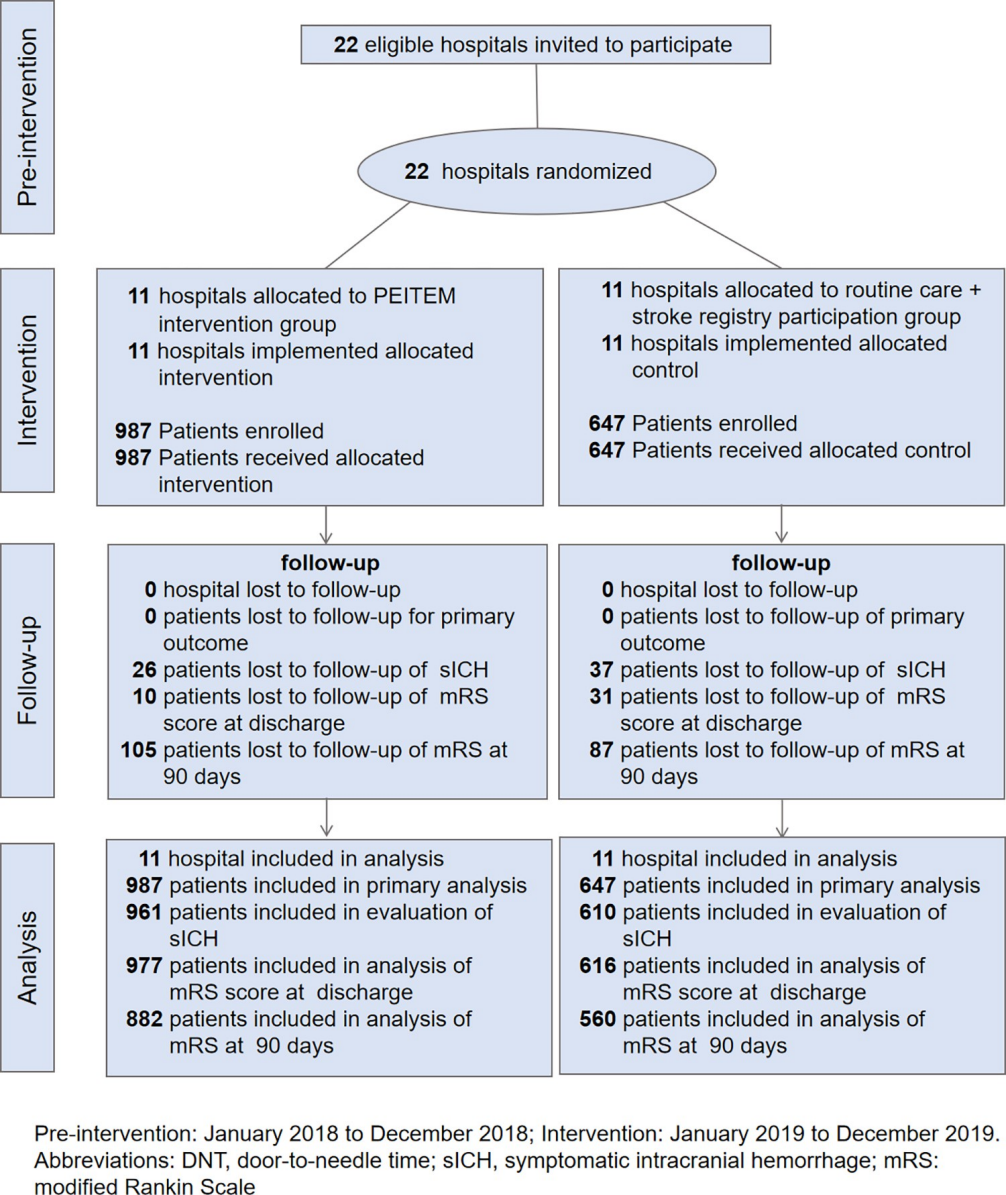
Flow of hospitals and patients through the study. mRS, modified Rankin scale; PEITEM, Persuasion Environment reconstruction Incentivization Training Education Modeling; sICH, symptomatic intracranial hemorrhage.

A total of 22 hospitals were included in the trial, and 1,634 patients were enrolled prospectively from January 1, 2019 through December 31, 2019 and included in the primary outcome analysis. Study design and timeframe are provided (**Fig 1**).

As **Table 1** shows, 81.8% of the participating hospitals were tertiary hospitals in China’s medical system and 72.7% of the hospitals had a stroke unit. During the preintervention period, a total of 1,363 AIS patients received IVT, including 724 (53.1%) thrombolytic patients in the PEITEM group and 639 (46.9%) thrombolytic patients in the control group. The average proportion of DNT ≤60 minutes in IVT patients was 62.0% versus 63.7% in the PEITEM group and control group, respectively. During the intervention period, the mean age of the included patients was 69 ± 13 years and 649 (39.7%) were female. The median baseline NIHSS was 6 (3 to 12). IVT was performed in 987/1,634 (60.4%) AIS patients in the PEITEM group and in 647/1,634 (39.6%) AIS patients in the control group. There were 4 (0.4%) patients in the PEITEM group and 3 (0.5%) in the control group with stroke mimic (*P* > 0.05). Patient characteristics were balanced between the PEITEM group and the control group, except for smoking, history of stroke/TIA, and prior antiplatelet usage. Baseline characteristics of patients with and without 90 days mRS measurement are shown (**Table B in S2 Text**).

**Table 1:** Baseline characteristics of the participating hospitals and patients with AIS implementing PEITEM intervention vs. routine care and stroke registry participation group (control) before and after implementation.

	Overall	PEITEM group	Control group
Hospital characteristics*			
Hospital, n	22	11	11
Patient, n	1,363	724	639
Hospital grade, n (%)			
Tertiary	18 (81.8)	10 (90.9)	8 (72.7)
Secondary	4 (18.2)	1 (9.1)	3 (27.3)
Stroke unit, n (%)	16 (72.7)	9 (81.8)	7 (63.6)
Teaching hospital, n (%)	18 (81.8)	8 (72.7)	10 (90.9)
No. of hospital beds, median (IQR)	1,075 (855–1,525)	1,150 (900–1,300)	955 (830–1,800)
No. of neurological ward beds, median (IQR)	62 (49–98)	63 (47–96)	61 (49–112)
Annual AIS discharges, median (IQR)	825 (575–1,200)	873 (800–1,200)	600 (480–1,500)
Preintervention DNT ≤60 minutes, n (%)	856/1,363 (62.8)	449/724 (62.0)	407/639 (63.7)
**Patient characteristics** †			
Patients, No. n	1,634	987	647
Demographics			
Age, (Mean ± SD), years	69 ± 13	69 ± 13	70 ± 12
Female, n (%)	649 (39.7)	411 (41.6)	238 (36.8)
Medical history			
Atrial fibrillation, n (%)	250 (15.3)	150 (15.2)	100 (15.5)
Coronary heart disease, n (%)	131 (8.0)	79 (8.0)	52 (8.0)
Hypertension, n (%)	1,054 (65.4)	642 (65.0)	412 (63.7)
Diabetes, n (%)	248 (15.2)	144 (14.6)	104 (16.1)
Smoking, n (%)	502 (30.7)	322 (32.6)	180 (27.8)
History of stroke/TIA, n (%)	189 (11.6)	128 (13.0)	61 (9.4)
Prior antiplatelet usage	227 (13.9)	151 (15.4)	76 (11.8)
SBP median (IQR), mm Hg,	155 (140–168)	156 (140–169)	154 (140–168)
DBP, median (IQR), mm Hg,	85 (77–93)	85 (77–93)	86 (77–94)
Prestroke mRS <2, n (%)	1,503 (92.0)	905 (91.7)	598 (92.4)
Baseline NIHSS, median (IQR)	6 (3–12)	6 (3–12)	6 (3–12)
Thrombectomy, n (%)	128 (7.8)	87 (8.8)	41 (6.3)

Value are mean ± SD, median (interquartile range), or No. (%) as appropriate. Wilcoxon rank-sum test and chi-squared test to compare the continuous variables and categorical variables, respectively.

*Data before implementation.

^†^Data after implementation.

AIS, acute ischemic stroke; DBP, diastolic blood pressure; DNT, door-to-needle time; IQR, interquartile range; mRS, modified Rankin scale score; NIHSS, National Institutes of Health Stroke Scale; TIA, transient ischemic attack; SBP, systolic blood pressure.

### Primary outcome

**Fig 2:**
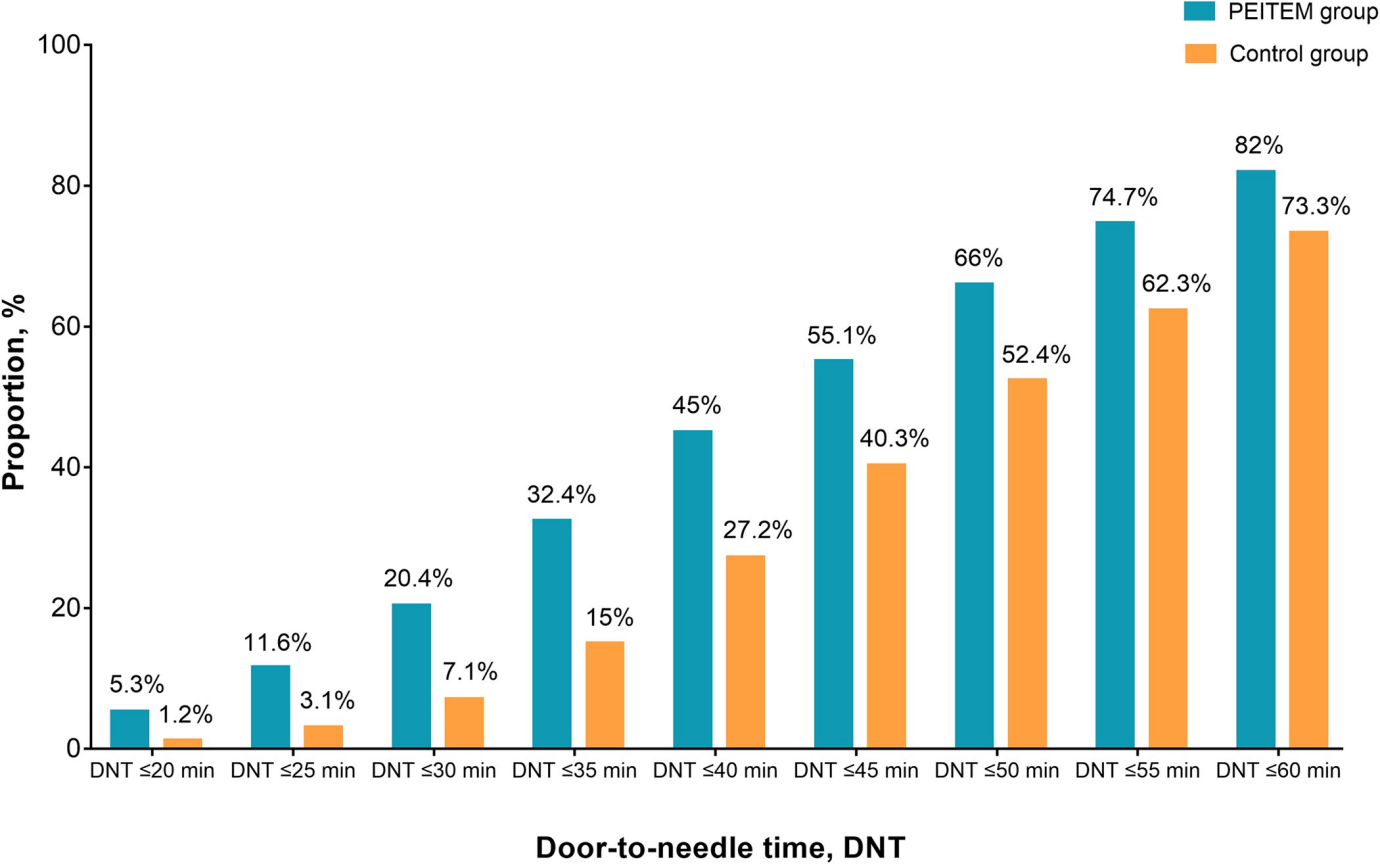
The proportion of AIS patients for different door-to-needle times in the PEITEM group and control group. AIS, acute ischemic stroke; DNT, door-to-needle time; PEITEM, Persuasion Environment reconstruction Incentivization Training Education Modeling.

The proportion of DNT ≤60 minutes in the PEITEM group was higher than in the control group after adjusting for patient and hospital characteristics (82.0% versus 73.3%; unadjusted odds ratio, 1.66; 95% confidence interval (CI), 1.31 to 2.11; adjusted odds ratio, 1.77; 95% CI, 1.17 to 2.70; ICC, 0.04; *P* = 0.007) (**Fig B in S2 Text and Table 2**). The proportion of AIS patients for different DNT in the PEITEM group and control group is presented in **Fig 2**.

**Table 2:** Door-to-needle time, functional outcomes and complications among eligible patients with acute ischemic stroke receiving PEITEM intervention vs. routine care and stroke registry participation group (control).

Variables	ICC	PEITEM group, No. of events/total patients (%)	Control group, No. of events/total patients (%)	Odds ratio (95% CI)	Mean difference (95% CI)	*P* value
** Primary outcome**						
DNT ≤60 minutes, n (%)*	0.04	809/987 (82.0)	474/647 (73.3)	1.76 (1.15, 2.70)	NA	0.009
** Secondary outcome**						
sICH, n (%)^†^	0.003	22/961 (2.3)	10/610 (1.6)	1.22 (0.49, 3.03)	NA	0.674
Favorable functional outcome at 90 days, n (%)^†^	0.01	490/882 (55.6)	282/560 (50.4)	1.38 (1.00, 1.90)	NA	0.049
Death at discharge, n (%)^†^	0.01	22/987 (2.2)	16/647 (2.5)	0.82 (0.33, 2.05)	NA	0.673
mRS at discharge, median (IQR)^†^	0.05	1 (0–4)	1 (0–4)	NA	−0.02 (−0.36, 0.32)	0.903
DNT, median (IQR), minutes*	0.12	43 (33–56)	50 (39–62)	NA	−8.83 (−14.06, −3.59)	0.001
ONT, median (IQR), minutes*	0.03	147 (107–194)	157 (109–199)	NA	−5.54 (−13.28, 2.20)	0.161

Value are median (interquartile range) or No. (%) as appropriate.

*Adjusted for patient characteristics (including age, female, history of stroke/TIA, hypertension, diabetes, atrial fibrillation, coronary heart disease, prior antiplatelet usage, thrombectomy, smoking, prestroke mRS score, and NIHSS score at admission) and hospital characteristics (including hospital grade (tertiary and secondary), stroke unit, teaching hospital status, and annual stroke discharge).

^†^Adjusted for age, female, history of stroke/TIA, prior antiplatelet usage, thrombectomy, smoking, prestroke mRS score, and NIHSS score at admission.

CI, confidence interval; DNT, door-to-needle time; ICC, intracluster correlation coefficient; IQR, interquartile range; mRS, modified Rankin scale; ONT, onset-to-needle time; sICH, symptomatic intracranial hemorrhage.

We also conducted a sensitivity analysis including all AIS patients treated with IVT within 4.5 hours and beyond 4.5 hours upon stroke onset during the intervention period. The proportion of DNT ≤60 minutes remained higher in the PEITEM group than in the control group (81.2% versus 72.3%; adjusted odds ratio, 1.81; 95% CI, 1.19 to 2.77, *P* = 0.006) (**Table C in S2 Text**).

### Secondary outcomes

The median DNT was 43 minutes in the PEITEM group and 50 minutes in the control group after adjusting for patient and hospital characteristics (adjusted mean difference: −8.83; 95% CI, −14.03 to −3.64; ICC, 0.12; *P* = 0.001). However, the median ONT was 147 minutes in the PEITEM group and 157 minutes in the control group, and the smaller ONT difference did not reach statistical significance (adjusted mean difference: −5.33; 95% CI, −13.29 to 2.63; ICC, 0.03; *P* = 0.189) (**Fig B in S2 Text and Table 2**).

**Fig 3:**
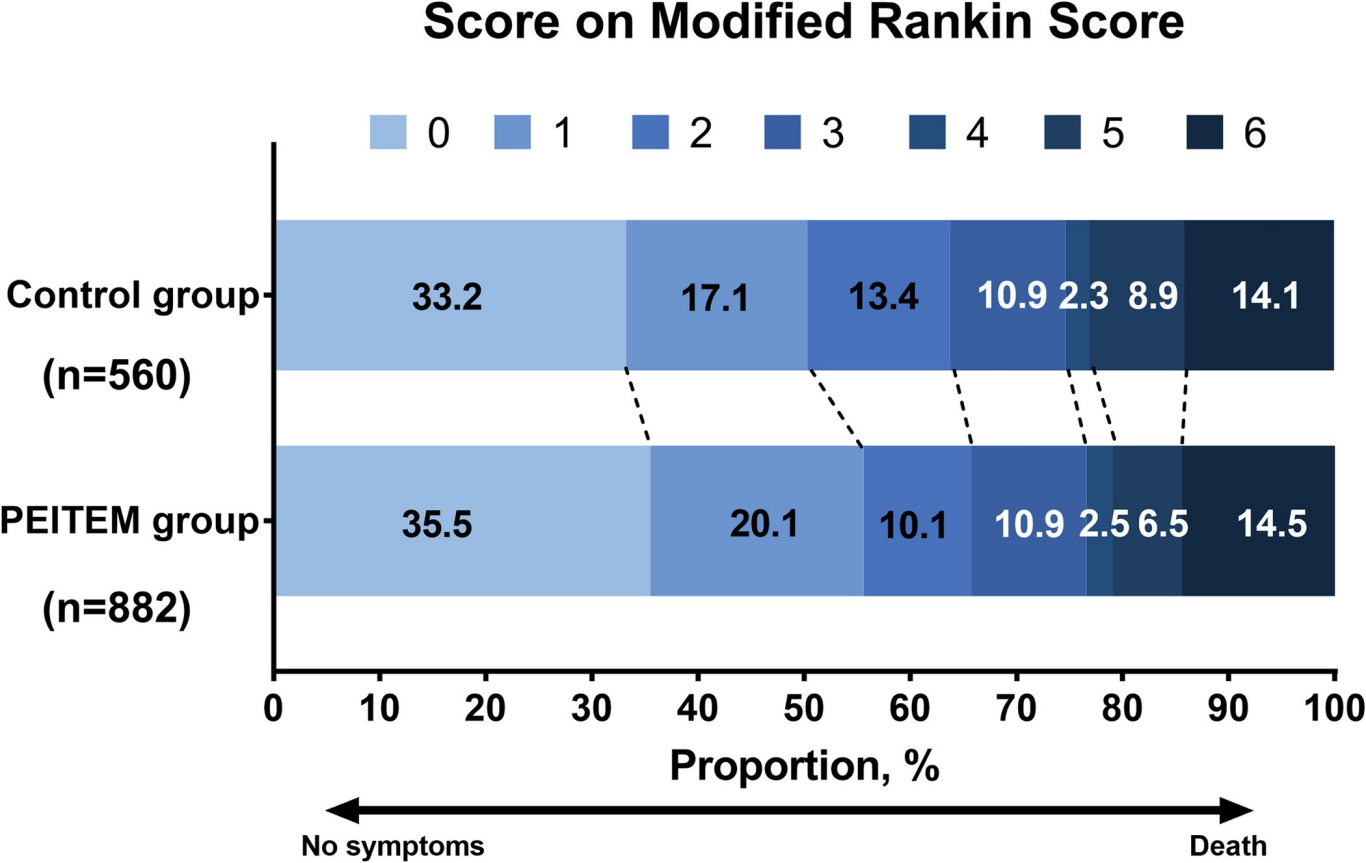
Distribution of modified Rankin scores at 90 days among eligible patients with AIS receiving PEITEM intervention vs. routine care and stroke registry participation group (control). AIS, acute ischemic stroke; PEITEM, Persuasion Environment reconstruction Incentivization Training Education Modeling.

Missing data were mainly due to the lack of brain imaging at 24 hours and the loss to the follow-up in the secondary outcome analysis (**Fig 1)**. Distribution of mRS score at 90 days is shown in **Fig 3**. The mRS values showed that 490 of 882 patients (55.6%) in the PEITEM group and 282 of 560 patients (50.4%) in the control group achieved 0 or 1 at 90 days, with an unadjusted absolute between-group difference of 5.2% (adjusted odds ratio, 1.38; 95% CI, 1.00 to 1.91; ICC, 0.01; *P* = 0.049). Moreover, favorable functional outcome at 90 days remained higher in the PEITEM group than in the control group after multiple imputation for missing data (adjusted odds ratio, 1.37; 95% CI, 1.01 to 1.85; *P* = 0.041). The median mRS score at discharge was 1 both in the PEITEM and control groups, and 22 of 961 patients (2.3%) in the PEITEM group and 10 of 610 (1.6%) patients in the control group had sICH at 24 hours after IVT. No significant between-group differences were found in mRS score at discharge (adjusted mean difference: −0.02; 95% CI, −0.36 to 0.32; ICC, 0.05; *P* = 0.903), the occurrence of sICH (adjusted odds ratio, 1.22; 95% CI, 0.49 to 3.03; ICC, 0.003; *P* = 0.674) and death at discharge (adjusted odds ratio, 0.82; 95% CI, 0.33 to 2.05; ICC, 0.01; *P* = 0.673) (**Fig B in S2 Text and Table 2**).

## Discussion

In the MISSION study, the teleconference-delivered multicomponent behavior intervention based on the BCW method was found to be effective in shortening DNT in IVT patients. Moreover, this intervention was associated with higher rate of favorable functional outcome in AIS patients in China.

Various approaches have been shown to improve DNT. A Norwegian stroke center implementing a revised treatment protocol reduced DNT from 27 to 13 minutes and improved patient outcomes in a quality improvement project [27]. However, their study is limited by the pre-post analysis. Actually, reducing DNT could be a complex clinical process, requiring coordination across departments and disciplines [28,29]. The improvement may vary across the hospital types, layouts, and regional polices. In 2010, AHA/ASA launched the “Target: Stroke,” which has increased the percentage of DNT less than 60 minutes from 26.5% to 41.3% in participating hospitals [24]. Note that the Target-Stroke endorsed 10 key strategies to reduce DNT in a survey of 304 hospitals, revealing the association between the use of a specific strategy and reduction in DNT [15]. Nevertheless, the quality improvement projects both in Norway and Target-Stroke were not randomized clinical trials, which may have led to an overestimation of the intervention effects. In contrast, our study was a cluster-randomized trial, which could better evaluate the effect of intervention on DNT, minimizing the potential biases.

The previous Thrombolysis Implementation in Stroke (TIPS) study in Australia used a combined multicomponent and multidisciplinary in-hospital approach based on BCW, but it showed no overall effect on DNT [30]. The underlying mechanism for the no effect is unknown. Nevertheless, it was conducted over a 5-year period from 2011 to 2015, when their national health system has implemented a number of health policies to improve stroke management. The influences from these policies might have already diluted the intervention’s effects in TIPS study. Indeed, in our MISSION study, the rate of DNT≤60 minutes was increased from 63.7% to 73.7% even in the control group from 2018 to 2019, as several strategies in China at the national level have been introduced to improve stroke care since 2018. Hence, the additional effect of intervention on DNT within 1 year in our study could have reflected the improvement of stroke service utilization at the hospital level.

We developed the PEITEM intervention to reduce DNT based on the known reasons leading to the thrombolytic delay. We identified at least 3 specific categories of such reasons from the database of 17,432 patients collected from the GWTG-Stroke registry: (1) the social reasons such as patient’s or family’s initial refusal, or their inability to determine; (2) the medical reasons such as hypertension management or concomitant emergent/acute condition; and (3) the hospital reasons such as the diagnostic delay, in-hospital delay, and/or equipment-related delay [31]. The PEITEM activities targeted each of these 3 categories. The Training and Education implementation can decrease the medical reasons and eligibility problems, while Persuasion and Modeling address the hospital reasons. Meanwhile, Environment Reconstruction and Incentivization complement all other interventions. Of note, our design and selection of the PEITEM intervention was based on the analysis of the behavioral nature, which is the core of the BCW method. The intervention mobilized the clinician’s capability, opportunity, and motivation to largely reduce IVT delay. Moreover, PEITEM intervention has taken full advantage of the advanced health information and internet technology, which might have prompted a novel and efficient model to improve medical quality.

In the current trial, patients in the PEITEM group had a significant higher rate of mRS 0 to 1 at 90 days after stroke onset. This finding is similar to a recent study from the GWTG hospitals, which demonstrated the association of shorter DNT with a lower all-cause mortality at 1 year, emphasizing the effects of the overall shortened DNT on functional outcome [32]. Data from China Stroke Statistics 2019 reported that there were 81.9% of ischemic stroke among stroke inpatients and 24.2% of them received IVT within 4.5 hours [9]. Given the increasing disease burden from stroke in the coming years, a large number of stroke patients might benefit from shortened DNT if quality improvement such as PEITEM intervention could be implemented in more stroke centers.

In the current study, we have successfully applied the BCW method to develop a component intervention to shorten DNT and confirm the usability and usefulness of the PEITEM intervention in clinicians’ behavior. Particularly, our PEITEM intervention delivered via video teleconference may be more appropriate and easier to implement in the current global Coronavirus Disease 2019 (COVID-19) public health crisis disrupting healthcare services.

Despite the robust improvement of clinical management, our study had several limitations. First, the group allocation could not be masked to the clinicians because of the intervention nature, although the balance in the baseline hospital characteristics decreased the bias. Second, the quality improvement intervention was studied over only 1-year period. Additional studies are needed to determine whether improvement in stroke care would be attained over a longer period. Third, the secondary analyses of mRS score at 90 days may subject to potential bias due to loss to follow-up. However, this influence was minimal in our study because of the comparable baseline characteristics among patients with and without the 90 days mRS measurements. Fourth, we did not have the data of patients who were not treated with IVT. It is unclear whether PEITEM intervention could improve the proportion of eligible patients who could have been treated. However, we do have the thrombolytic rate within 7 days of AIS patients, that is, the total number of patients receiving IVT divided by the total number of patients within 7 days of symptom onset. The thrombolytic rate within 7 days increased from 8.8% to 13.2% in the PEITEM group after intervention, while it decreased from 11.0% to 9.3% in the control group. Fifth, the intervention might influence which patients receive IVT. There might be residual measured and unmeasured confounders that may influence DNT and outcomes although multiple patient-level and hospital-level baseline characteristics were adjusted in outcome analyses. Sixth, patients with stroke mimic were not excluded from the current study although there were only 7 patients with stroke mimic. At the last, we implemented 6 interventions concurrently rather than testing individual intervention’s effect. Although we were able to conclude that the combining intervention can shorten DNT, we cannot infer whether any one of the individual PEITEM interventions would have the same effect.

## Conclusions

Our PEITEM intervention via a teleconference-delivered program resulted in a robust increase in the proportion of patients receiving rapid IVT within 60 minutes, leading to a moderate but clinically relevant shorter DNT and better functional outcomes at 90 days after onset in stroke patients. Our findings provide a novel avenue to improve stroke care quality for improving stroke treatment, which is meaningful for other developing countries, especially in the current global COVID-19 pandemic crisis.

## Supporting information

S1 Checklist CONSORT checklist. (DOCX)

S1 Text Protocol. (DOCX)

S2 Text Supporting Figure and Table. **Fig A.** PEITEM intervention diagram and its relationship with BCW methods. **Fig B.** Door-to-needle time, functional outcome and complication among eligible patients with acute ischemic stroke receiving PEITEM intervention vs. routine care and stroke registry participation group (Control). **Table A.** Description of PEITEM intervention according to the Behavior Change Wheel components. **Table B.** Baseline characteristics between patients with and without the modified Rankin scale at 90 days. **Table C.** Door-to-needle time, functional outcome and complication among patients who received IVT during the intervention period between PEITEM and control group for sensitivity analysis.
(DOCX)

## Abbreviations

AIS: acute ischemic stroke
AHA/ASA: American Heart Association/American Stroke Association
BCW: Behavior Change Wheel
CI: confidence interval
COVID-19: Coronavirus Disease 2019
CT: computed tomography
DNT: door-to-needle time
ICC: intracluster correlation coefficient
IVT: intravenous thrombolysis
mRS: modified Rankin scale
NIHSS: National Institutes of Health Stroke Scale
ONT: onset-to-needle time
PEITEM: Persuasion Environment reconstruction Incentivization Training Education Modeling
QCI: quality care initiative
sICH: symptomatic intracranial hemorrhage
TIPS: Thrombolysis Implementation in Stroke
