# Supplementary material for: Evaluation of a multicomponent intervention to shorten thrombolytic door-to-needle time in stroke patients in China (MISSION): A cluster-randomized controlled trial
Source: PLoS Med. 2022 Jul 5;19(7):e1004034. doi: 10.1371/journal.pmed.1004034 (PMC9255731; doi:10.1371/journal.pmed.1004034)
Supplement: S1 Text — (DOCX) [file pmed.1004034.s002.docx]

**Improving in-hospital stroke service utilization to** **shorten thrombolytic door-to-needle time in patients with acute ischemic stroke in China (MISSION): a cluster randomized controlled trial**

**Local Investigators and Clusters Participating in this trial**

The Second Hospital of Shaoxing City;

People’s Hospital of Haining City;

People’s Hospital of Haiyan County;

Huzhou No.1 People’s Hospital;

Jiashan County No.1 People’s Hospital;

Ningbo Medical Center Lihuili Hospital;

Tongxiang No.1 People’s Hospital;

The First Affiliated Hospital of Wenzhou Medical University;

Central Hospital of Yiwu City;

Yongkang No.1 People’s Hospital;

People’s Hospital of Yuyao City;

People’s Hospital of Deqing County;

People’s Hospital of Dongyang City;

People’s Hospital of Jinhua City;

People’s Hospital of Lishui City;

Central Hospital of Lishui City;

People’s Hospital of Lin’an City;

People’s Hospital of Longquan County;

People’s Hospital of Qingtian County;

Central Hospital of Shaoxing City;

Central Hospital of Wenzhou City;

Xiangshan County No.1 People’s Hospital.

**Introduction**

Intravenous thrombolysis (IVT) reduces long term disability when administered early to eligible patients with acute ischemic stroke (AIS), but its benefit is strongly time-dependent.^1-4^ Moreover, earlier administration of IVT, compared with later administration, has been shown to be associated with lower risk of in-hospital mortality and hemorrhagic transformation, and better functional outcomes at discharge and at 90 days^5-7^. The AHA/ASA guidelines thus recommend to initiate tissue plasminogen activator (tPA) treatment within 60 minutes after the patient’s arrival in the emergence department.^8^ However, a cohort analysis of 1193 patients in the United States found a median DNT of 82.9 minutes.^9^ The Target: Stroke initiative was launched in 2010 to assist hospitals in providing tPA in a timely fashion. As a result, the percentage of alteplase-treated patients having door-to-needle time (DNT) of ≤60 minutes increased from 26.5% to 41.3%.^10^ In single large metropolitan comprehensive stroke centers (Helsinki and Melbourne), major reduction in DNT has been reported with multifaceted intervention.^11, 12^

[Particularly](C:/Users/Administrator/AppData/Local/youdao/dict/Application/8.9.3.0/resultui/html/index.html#/javascript:;), the delay of IVT is serious in China. Data from Chinese National Stroke Registry (CNSR) showed that the median DNT was 116 minutes, while only 17.8% of patients were treated with IVT within 60 minutes after admission.^13^ The time in CNSR was longer than those in SITS-MOST Ethos and Registry of SITS-EAST.^14, 15^ Moreover, data from the ENCHANTED indicated that Chinese patients were twice as likely as non-Chinese patients to have DNT > 60 minutes.^16^ A comprehensive program is clearly demanded in China, with ongoing monitoring of DNT as an important benchmark for quality in stroke care.

Although reducing in-hospital delay has been one of the focuses of stroke quality control in China, there is a lack of clinical trials to confirm the effectiveness of clinical interventions to reduce in-hospital delay. Improving In-hospital Stroke Service Utilisation (MISSION) study thus aimed to explore whether a multi-component behavior change intervention based on Behaviour Change Wheel (BCW) method via video teleconference could shorten DNT in China.

**Study objective**

The aim is to evaluate whether a multi-component intervention via video teleconference based on the BCW method increases the proportion of DNT ≤ 60 minutes in AIS treated with IVT.

**Design**

The MISSION is an open label, multicenter cluster-randomised clinical trial involving 22 hospitals in China. The Zhejiang Stroke Alliance developed by Zhejiang Stroke Quality Control Center (ZSQCC) has consecutively recruited stroke patients in eastern China since 2016. This trial was approved by the human ethics committee of the Second Affiliated Hospital of Zhejiang University (SAHZU), School of Medicine. The clinical trial is conducted according to the principle expressed in the Declaration of Helsinki. Written informed consent will be obtained from the participating hospital staff. Informed consent will also be obtained from patients or their legally authorized representatives for treatment with alteplase and participation in a telephone survey at 3, 6, and 12 months after the initial stroke accordance with the local hospital standards.

The clinicians in one group of hospitals will receive the PEITEM intervention, a multi-components behavior change intervention based on the behavioral principles, as described by the Behavior Change Wheel framework, whereas the clinicians in another group of hospitals as the control will only receive routine care plus stroke registry. Study activities will be divided into two periods: the Pre-intervention (January 2018 to December 2018) and the Intervention (January 2019 to December 2019). Fig A describes the study design.

**Participants: study clusters and their patients**

In China, hospitals are classified into 3 grades: community hospitals are defined as primary grade, hospitals that serve several communities are defined as secondary

grade, and central hospitals for a certain district or city are defined as tertiary grade. Only secondary or tertiary public hospitals with emergency departments (EDs) and neurological wards that admit patients with stroke and had the capacity to administer intravenous rtPA were eligible to participate in this trial. Hospitals participating in the Zhejiang Stroke Alliance meeting the inclusion and exclusion criteria are eligible for inclusion in the MISSION trial. Hospitals meeting the following criteria are eligible for the study: (1) agree to participate in the ongoing data report and continuous audit of IVT processes of care and outcomes; (2) have a stroke unit or staffing equivalent to a stroke physician and a nurse. The hospitals with fewer than 20 thrombolytic cases per year will be excluded. Only the AIS patients receiving IVT within 4.5 hours are eligible for inclusion.

**Clusters randomization and blinding**

Because the hospitals are the intervention targets, they are used as the randomization unit to minimize contamination. The hospitals will be stratifically randomized with a 1:1 allocation ratio according to their baseline proportion of IVT patients with DNT ≤60 minutes (<60%, ≥ 60% -≤ 72%, >72%) collected in the pre-intervention period. The randomization will be conducted by using a computer generated randomisation sequence, by which eleven hospitals are assigned to the intervention group and another eleven hospitals to the control group. Masking the hospital personnel and clinical investigators to site or group assignment is not possible because of the intervention nature. Nevertheless, external clinical evaluators assigned to assess the outcomes will be masked.

**Data monitoring and quality control**

Data on all thrombolytic patients in both the PEITEM and control group will be recorded in a secure, purpose-built web-based data entry system. The system provides pre-defined logic checks to identify erroneous, illogical data entries. All patient data will be de-identified and entered by a local trained study-specific delegate who is independent from the study team at each participating hospital. The database is only accessible via a secure login system and only accessible to those approved to do so. Moreover, only the de-identified documents are preserved and monitored by an independent contract research organization throughout the study period. All recruitment will be checked by research specialists from an independent contract research organization (CRO) throughout the study period. Finally, a data quality meeting will be held quarterly to review all the hospital records and registry data.

Data will include the detailed eligibility assessment and demographics, National Institutes of Health Stroke Scale (NIHSS) score, prestroke modified Rankin Scale (mRS) score, risk factors, hemorrhagic transformation, baseline blood pressure, prior antiplatelet usage, DNT, onset-to-needle time (ONT), modified Rankin Scale (mRS) score at discharge and 90 days. mRS score at 90 days of AIS patients will be followed up with telephone questionnaires by external clinical evaluators who are blinded to the patients’ clinical data.

**Trial management and monitoring**

The Trial Management Group (TMG) will be responsible for all aspects of local organization and the day-to-day running of the trial, including monitoring outcome data collection, and ensuring the implementation of PEITEM intervention to the participating hospitals. An independent Trial Steering Committee (TSC) will oversee the trial, and they will meet at least monthly to consider and address strategic issues. Moreover, a training physician from the department of neurology in each cluster will act as a quality coordinator. The quality coordinator’s role includes interacting with physicians once gaps in the PEITEM intervention are identified, identifying barriers for the implementation and ensuring that all the intervention are implemented. There will be a run-in period before the start of the trial. The TMG will have regular meetings with quality coordinators of the PEITEM hospitals to discuss the detail of the video conferences and to assess the infrastructures to ensure that the intervention programs can be carried out regularly after the trial begins. The evaluation of outcome will start after the run-in period. The member of a Data Monitoring Committee (DMC) will act independently of the TSC, TMG and funder, will monitor data, check the data quality submitted by the study-specific delegate, and make recommendations to the TSC on whether there are any ethical or safety reasons, if exist, why the trial should not continue. Deaths by any cause in the 1 year after hospitalization, the Serious Adverse Events (SAEs), will be recorded and reported in this trial. The DMC will monitor the occurrence of SAEs.

**Intervention**

Hospitals assigned to the PEITEM group will implement the PEITEM intervention from January 1, 2019 through December 31, 2019. The PEITEM intervention components are grounded in the behavioral theory in the Behavior Change Wheel (BCW) method.^17^ The BCW emphasizes the importance of ensuring that proponents have the capability, opportunity and motivation to perform the desired behavior through nine interventions. The PEITEM intervention will implement six of them including **P**ersuasion, **E**nvironmental reconstruction, **I**ncentivization, **T**raining, **E**ducation and **M**odeling.

*Persuasion and Modeling* aim to foster communication between clinicians and research team to stimulate clinician’s action to shorten DNT and to provide a good example to follow. A series of measures are embedded such as setting up DNT target, time tracking with feedback, case discussion and championship experience presentation to decrease in-hospital delay. *Training and Education* can help to improve the skills and knowledge of the clinicians. The Education involve the early identification of eligible patients for IVT, rapid decision-making based on the rapid risk evaluation of hemorrhagic transformation and complication management, and the training of sustainable application of quality improvement tools; the purpose will be to help rapid diagnosis, decrease initial refusal and improve complication management. *Incentivization* encouraged stroke doctors to create expectation of reward. We will establish a pioneer award according to the number of IVT patients and the proportion of DNT ≤ 60min. Finally, we will implement the standardized medical record templates involving evidence-based performance measures to indicate *Environment reconstruction*, as a persist intervention, aiming to change the physical context of patients’ information collection. A summary of the PEITEM intervention and its link with the BCW is shown in **Fig B** and a detailed description is given in **Table A.**

The PEITEM intervention will be implemented by a professional medical quality care initiative (QCI) team. Stroke team and emergency department team of intervention group will attend a 2-hour video tele-conference monthly. Training video or slides will be provided to the clinicians who can not attend the video tele-conference on time. Each hospital in the intervention group will provide formal documentation of completed training such as photos and attendance logs to guarantee adequate knowledge translation on the implementation of each intervention to the QCI team.

**Outcomes**

The primary outcome is the proportion of DNT ≤ 60 min in the AIS treated with IVT, indicating a fast thrombolytic treatment from hospital arrival. The secondary outcomes include the key time intervals between assessment and thrombolytic treatment (median DNT, ONT), mRS score and death at discharge, complications after IVT [symptomatic intracranial hemorrhage (sICH)] and favorable functional outcomes (score of 0 to 1 on the mRS at 90 days), which indicate the efficiency and safety of PEITEM intervention. The DNT is defined as the time between hospital arrival and the initiation of IVT. The ONT is defined as the time between the symptom onset and the initiation of IVT. sICH is defined as intracranial hemorrhage at 24 hours associated with an increase of ≥4 points of NIHSS score from baseline, according to European Cooperative Acute Stroke Study (ECASS) II trial.^18^

**Sample size**

A pre-randomization survey in the pre-intervention period at participating clusters was conducted. The proportion of AIS patients receiving IVT with DNT ≤ 60 minutes in China was 62%. Based on the outcomes reported by previous studies, a sample size estimation with a change from 55% to 70% of patients with DNT ≤ 60 minutes is clinically important.^10^ Therefore, a total of 1424 patients at 22 hospitals will be required to detect a 15% improvement in AIS patients with DNT ≤ 60 minutes, with 80% power, 5% significance level, and an intracluster correlation coefficient (ICC) of 0.05.

**Statistical Analysis Plan**

We will use the intention-to-treat analysis to analyze all outcomes. The baseline characteristics of hospitals and patients will be analyzed between the PEITEM and control groups. We will summarize continuous variables as median with interquartile ranges and categorical variables as frequency and percentage, and analyze them by using Wilcoxon rank-sum test and Chi-square test separately. Modes will be used to impute missing values of categorical variables, and medians will be imputed for missing values of continuous variables.

Intra-cluster correlation coefficients (ICC) will be calculated using the correlation-based estimation methods for categorical outcomes and analysis of variance methods for continuous variables, respectively.^19^ Generalized estimating equation models with an exchangeable working correlation structure will be conducted to account for the correlations of observations within clusters (hospitals).^20^ Odds ratios for the binary outcomes (DNT ≤ 60 minutes, sICH, favorable functional outcome at 90 days, and death at discharge) and mean differences for the continuous outcomes (DNT, ONT, and mRS score at discharge) will be estimated to assess the treatment effects. Multivariable model for DNT ≤ 60 minutes, DNT, and ONT will be adjusted for patient characteristics (including age, sex, history of stroke/TIA, hypertension, diabetes, atrial fibrillation, coronary heart disease, prior antiplatelet usage, thrombectomy, smoking, prestroke mRS score, and NIHSS score at admission) and hospital characteristics (including hospital grade [tertiary and secondary], stroke unit, teaching hospital status, and annual stroke discharge). Clinical outcomes will be adjusted for age, pre-stroke mRS score, NIHSS score at admission, and other patient characteristics with a p value of <0.1 in the univariate analysis.

Additionally, to assess whether the improvement in primary and secondary outcomes in the PEITEM group is influenced by the time window, we will conduct a sensitivity analysis with the additional inclusion of patients receiving IVT beyond 4.5 hours upon stroke onset. All patients receiving IVT beyond 4.5 hours upon stroke onset should have CT or MRI core/perfusion mismatch. For patients lost to follow-up at 90 days, we will use multiple imputation as a sensitivity analysis and compared the baseline characteristics between patients with and without mRS at 90 days. A two-tailed P < 0.05 is considered statistically significant. All analyses will be performed using SAS (version 9.4) and R software (version 4.0.5). As no detrimental problems to the participant are anticipated, there are no formal stopping rules for the trial. This trial is registered with ClinicalTrials.gov, number NCT03317639.

**Fig A** Study design and timeframe.

**
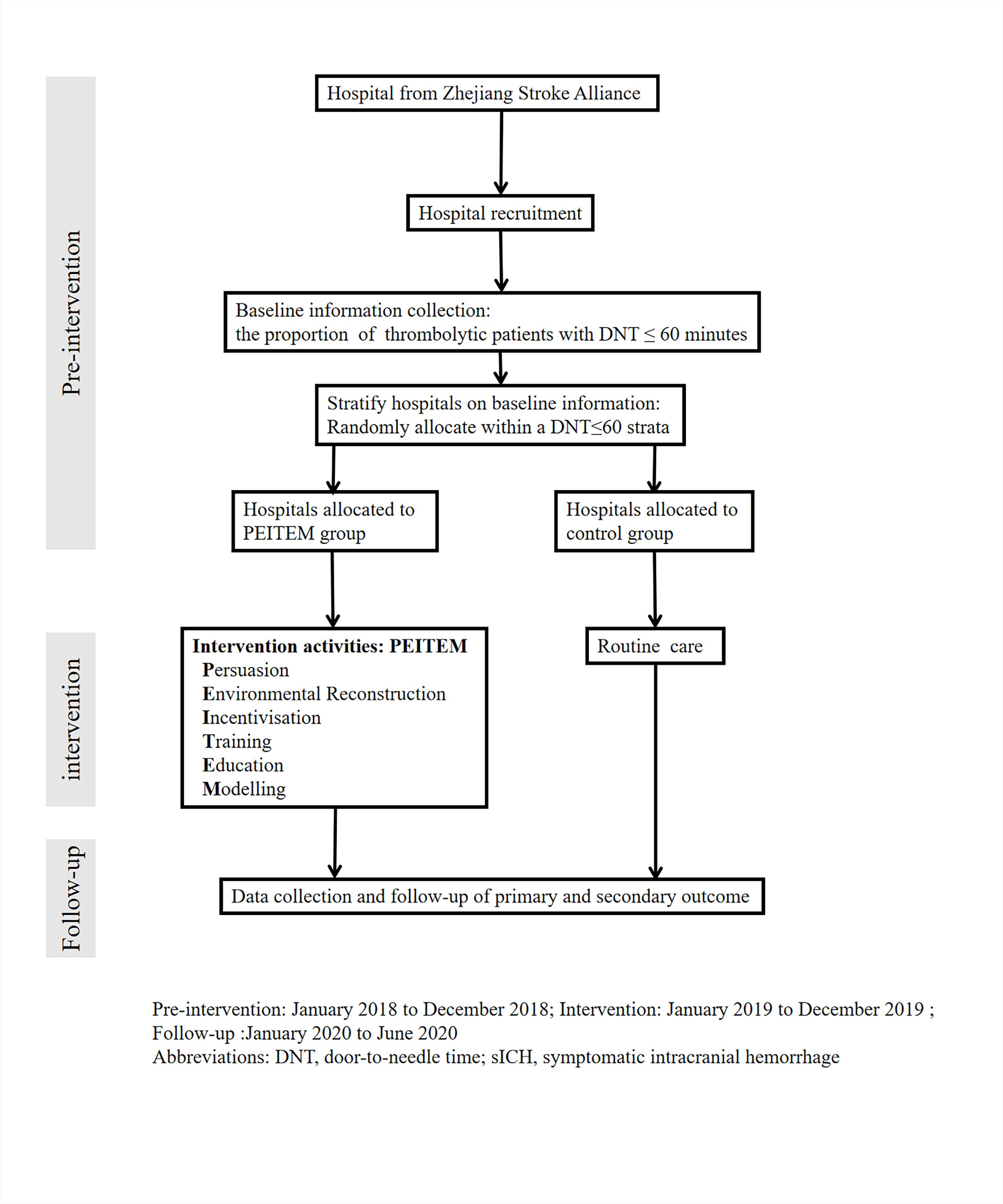
**

**Fig B** A PEITEM intervention diagram and its relationship with the BCW methods.


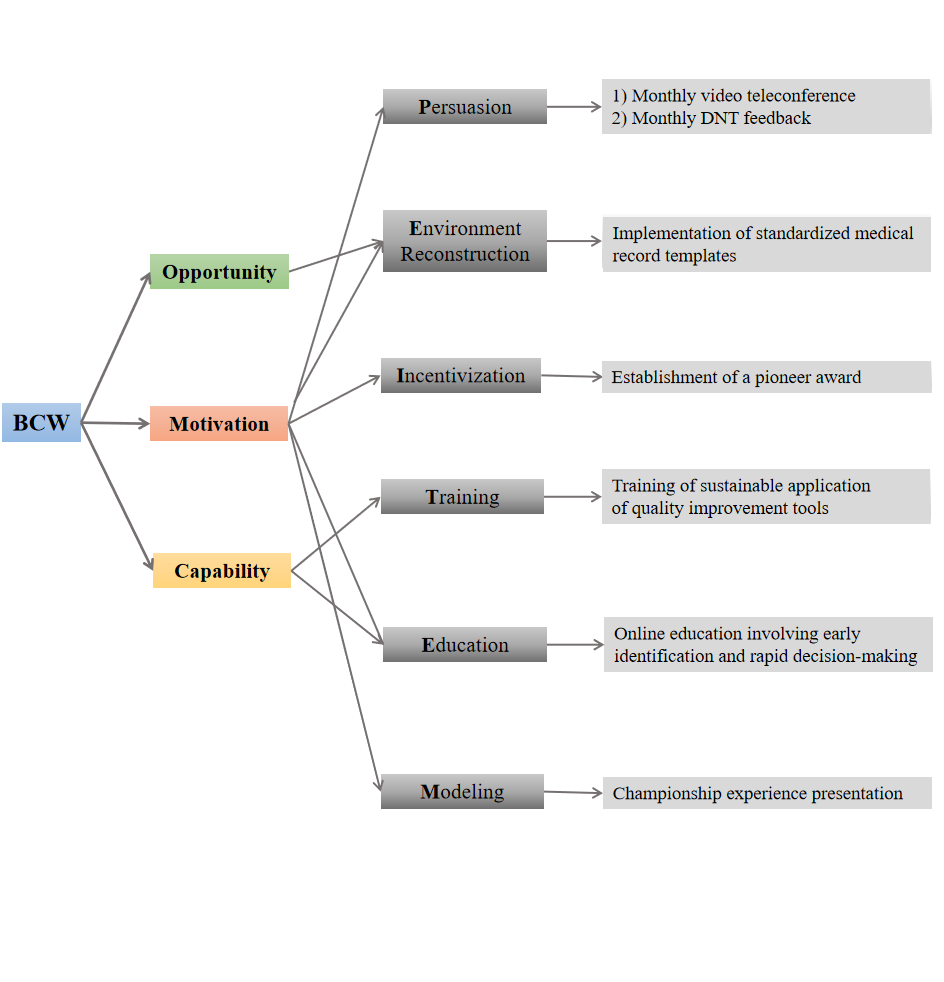


Abbreviations: BCW, Behaviour Change Wheel; DNT, Door-to-Needle Time.

**Table A** Description of PEITEM Intervention according to the Behavior Change Wheel Components.

| Intervention | Definition | Activity |
| --- | --- | --- |
| **P**ersuasion | Using communication to induce positive or negative feelings or stimulate action | 1. Monthly video teleconference between stroke doctors from PEITEM hospitals and research team for face-to-face problem solving; 2. Monthly DNT feedback by setting up DNT target, time tracking with feedback, discussion about the reasons of delay and improving measures through case discussion. |
| **E**nvironment Reconstruction | Changing the physical or social context | Implementation of standardized medical record templates involving evidence-based performance measures in the electronic medical record. |
| **I**ncentivization | Creating expectation of reward | Establishment of a pioneer award of IVT to the hospitals according to the number of IVT patients and the proportion of DNT ≤60min by research team. |
| **T**raining | Imparting skills | Training of sustainable application of quality improvement tools to help hospitals to establish their own personalized, efficient and stable code stroke workflow via video teleconference. |
| **E**ducation | Increasing knowledge or understanding | Education involving early identification of eligible patients for IVT, and rapid decision-making based on rapid risk evaluation of hemorrhagic transformation and complication management such as hypertension and hyperglycemia via video teleconference. |
| **M**odeling | Providing an example for people to aspire or imitate | Championship experience presentation by the PEITEM hospital with shortest DNT via video teleconference monthly. |

Abbreviations: DNT, Door-to-Needle Time; IVT, Intravenous thrombolysis.

**Reference**

1. Powers WJ, Rabinstein AA, Ackerson T, Adeoye OM, Bambakidis NC, Becker K, et al. 2018 guidelines for the early management of patients with acute ischemic stroke: A guideline for healthcare professionals from the american heart association/american stroke association. *Stroke*. 2018;49:e46-e110

2. Wardlaw JM, Murray V, Berge E, del Zoppo G, Sandercock P, Lindley RL, et al. Recombinant tissue plasminogen activator for acute ischaemic stroke: An updated systematic review and meta-analysis. *Lancet*. 2012;379:2364-2372

3. Lees KR, Bluhmki E, von Kummer R, Brott TG, Toni D, Grotta JC, et al. Time to treatment with intravenous alteplase and outcome in stroke: An updated pooled analysis of ecass, atlantis, ninds, and epithet trials. *Lancet*. 2010;375:1695-1703

4. Marler JR, Tilley BC, Lu M, Brott TG, Lyden PC, Grotta JC, et al. Early stroke treatment associated with better outcome: The ninds rt-pa stroke study. *Neurology*. 2000;55:1649-1655

5. Emberson J, Lees KR, Lyden P, Blackwell L, Albers G, Bluhmki E, et al. Effect of treatment delay, age, and stroke severity on the effects of intravenous thrombolysis with alteplase for acute ischaemic stroke: A meta-analysis of individual patient data from randomised trials. *Lancet*. 2014;384:1929-1935

6. Lansberg MG, Schrooten M, Bluhmki E, Thijs VN, Saver JL. Treatment time-specific number needed to treat estimates for tissue plasminogen activator therapy in acute stroke based on shifts over the entire range of the modified rankin scale. *Stroke*. 2009;40:2079-2084

7. Saver JL, Fonarow GC, Smith EE, Reeves MJ, Grau-Sepulveda MV, Pan W, et al. Time to treatment with intravenous tissue plasminogen activator and outcome from acute ischemic stroke. *JAMA*. 2013;309:2480-2488

8. Jauch EC, Saver JL, Adams HP, Jr., Bruno A, Connors JJ, Demaerschalk BM, et al. Guidelines for the early management of patients with acute ischemic stroke: A guideline for healthcare professionals from the american heart association/american stroke association. *Stroke*. 2013;44:870-947

9. Albers GW, Bates VE, Clark WM, Bell R, Verro P, Hamilton SA. Intravenous tissue-type plasminogen activator for treatment of acute stroke: The standard treatment with alteplase to reverse stroke (stars) study. *JAMA*. 2000;283:1145-1150

10. Fonarow GC, Zhao X, Smith EE, Saver JL, Reeves MJ, Bhatt DL, et al. Door-to-needle times for tissue plasminogen activator administration and clinical outcomes in acute ischemic stroke before and after a quality improvement initiative. *JAMA*. 2014;311:1632-1640

11. Meretoja A, Strbian D, Mustanoja S, Tatlisumak T, Lindsberg PJ, Kaste M. Reducing in-hospital delay to 20 minutes in stroke thrombolysis. *Neurology*. 2012;79:306-313

12. Meretoja A, Weir L, Ugalde M, Yassi N, Yan B, Hand P, et al. Helsinki model cut stroke thrombolysis delays to 25 minutes in melbourne in only 4 months. *Neurology*. 2013;81:1071-1076

13. Wang Y, Liao X, Zhao X, Wang DZ, Wang C, Nguyen-Huynh MN, et al. Using recombinant tissue plasminogen activator to treat acute ischemic stroke in china: Analysis of the results from the chinese national stroke registry (cnsr). *Stroke*. 2011;42:1658-1664

14. Wahlgren N, Ahmed N, Davalos A, Ford GA, Grond M, Hacke W, et al. Thrombolysis with alteplase for acute ischaemic stroke in the safe implementation of thrombolysis in stroke-monitoring study (sits-most): An observational study. *Lancet*. 2007;369:275-282

15. Harsany M, Kadlecova P, Svigelj V, Korv J, Kes VB, Vilionskis A, et al. Factors influencing door-to-imaging time: Analysis of the safe implementation of treatments in stroke-east registry. *J Stroke Cerebrovasc Dis*. 2014;23:2122-2129

16. Yang J, Wang X, Yu JP, Hang J, Lavados P, Robinson T, et al. Positive impact of the participation in the enchanted trial in reducing door-to-needle time. *Sci Rep*. 2017;7:14168

17. Michie S, van Stralen MM, West R. The behaviour change wheel: A new method for characterising and designing behaviour change interventions. *Implement Sci*. 2011;6:42

18. Hacke W, Kaste M, Fieschi C, von Kummer R, Davalos A, Meier D, et al. Randomised double-blind placebo-controlled trial of thrombolytic therapy with intravenous alteplase in acute ischaemic stroke (ecass ii). Second european-australasian acute stroke study investigators. *Lancet*. 1998;352:1245-1251

19. Chakraborty H, Hossain A. R package to estimate intracluster correlation coefficient with confidence interval for binary data. *Comput Methods Programs Biomed*. 2018;155:85-92

20. Leyrat C, Morgan KE, Leurent B, Kahan BC. Cluster randomized trials with a small number of clusters: Which analyses should be used? *Int J Epidemiol*. 2018;47:321-331
