# Supplementary material for: Evaluation of a multicomponent intervention to shorten thrombolytic door-to-needle time in stroke patients in China (MISSION): A cluster-randomized controlled trial
Source: PLoS Med. 2022 Jul 5;19(7):e1004034. doi: 10.1371/journal.pmed.1004034 (PMC9255731; doi:10.1371/journal.pmed.1004034)
Supplement: S2 Text — Fig A. PEITEM intervention diagram and its relationship with BCW methods. Fig B. Door-to-needle time, functional outcome and complication among eligible patients with acute ischemic stroke receiving PEITEM intervention vs. routine care and stroke registry participation group (Control). Table A. Description of PEITEM intervention according to the Behavior Change Wheel components. Table B. Baseline characteristics between patients with and without the modified Rankin scale at 90 days. Table C. Door-to-needle time, functional outcome and complication among patients who received IVT during the intervention period between PEITEM and control group for sensitivity analysis. (DOCX) [file pmed.1004034.s003.docx]

**Supplemental material**

**Fig A** PEITEM intervention diagram and its relationship with BCW methods.


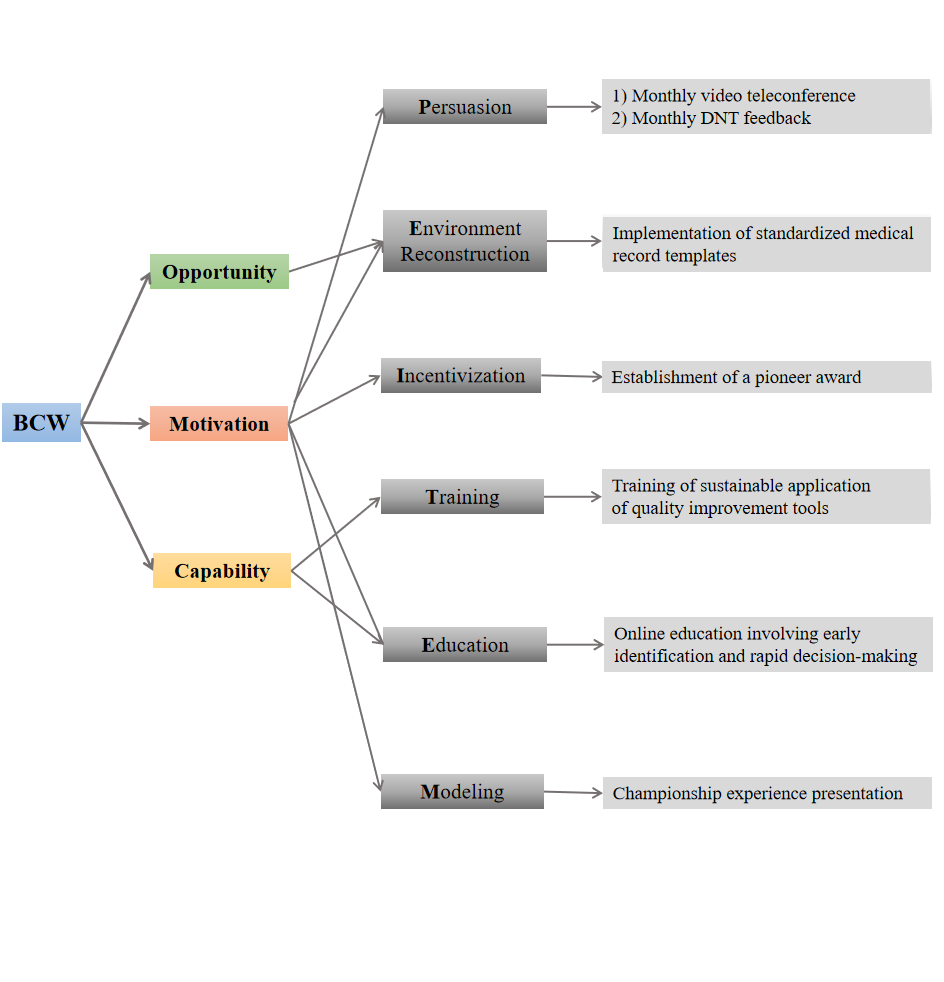
Abbreviations: BCW, Behaviour Change Wheel; DNT, Door-to-Needle Time.

**Fig B** Door-to-needle time, Functional Outcome and Complication Among Eligible Patients With Acute Ischemic Stroke Receiving PEITEM intervention vs Routine Care and Stroke Registry Participation Group (Control).


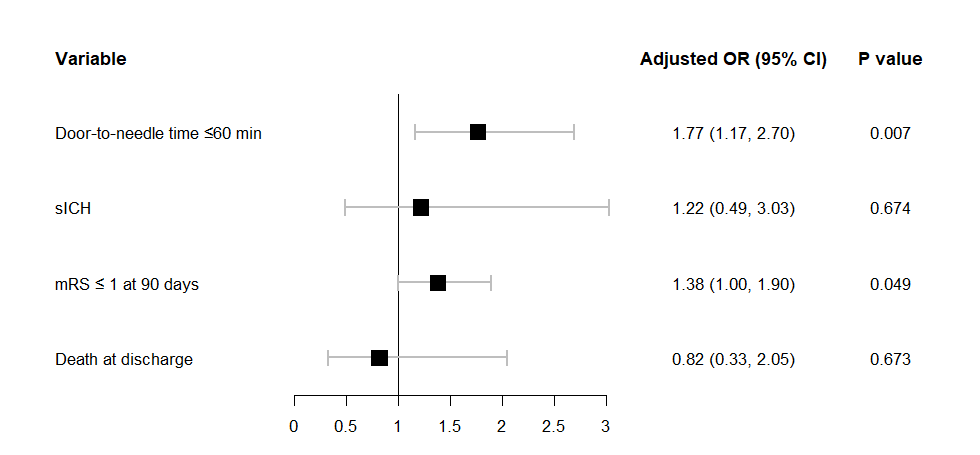


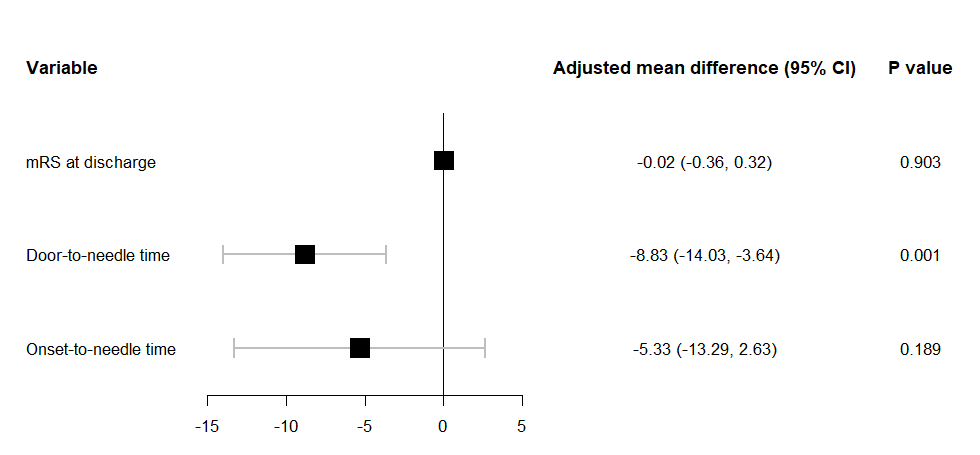


Abbreviations: OR, Odds Ratio; sICH, symptomatic intracranial hemorrhage; mRS, modified Rankin Scale.

**Table A** Description of PEITEM Intervention according to the Behavior Change Wheel Components.

| Intervention | Definition | Activity |
| --- | --- | --- |
| **P**ersuasion | Using communication to induce positive or negative feelings or stimulate action | 1. Monthly video teleconference between stroke doctors from PEITEM hospitals and research team for face-to-face problem solving; 2. Monthly DNT feedback by setting up DNT target, time tracking with feedback, discussion about the reasons of delay and improving measures through case discussion. |
| **E**nvironment Reconstruction | Changing the physical or social context | Implementation of standardized medical record templates involving evidence-based performance measures in the electronic medical record. |
| **I**ncentivization | Creating expectation of reward | Establishment of a pioneer award of IVT to the hospitals according to the number of IVT patients and the proportion of DNT ≤60min by research team. |
| **T**raining | Imparting skills | Training of sustainable application of quality improvement tools to help hospitals to establish their own personalized, efficient and stable code stroke workflow via video teleconference. |
| **E**ducation | Increasing knowledge or understanding | Education involving early identification of eligible patients for IVT, and rapid decision-making based on rapid risk evaluation of hemorrhagic transformation and complication management such as hypertension and hyperglycemia via video teleconference. |
| **M**odeling | Providing an example for people to aspire or imitate | Championship experience presentation by the PEITEM hospital with shortest DNT via video teleconference monthly. |

Abbreviations: DNT, Door-to-Needle Time; IVT, Intravenous thrombolysis.

**Table B**. Baseline Characteristics between Patients with and without the modified Rankin Scale at 90 days.

| **Variables** | **Patients with mRS**  **(n=1442)** | **Patients without mRS (n=192)** | ***P* value** |
| --- | --- | --- | --- |
| **Demographics** |  |  |  |
| Age, (Mean ± SD), years | 69 ± 13 | 69 ± 13 | 0.53 |
| Female, n (%) | 585 (40.6) | 64 (33.3) | 0.06 |
| **Medical history** |  |  |  |
| Atrial fibrillation, n (%) | 221 (15.3) | 29 (15.1) | >0.99 |
| [Coronary](C:/Users/Administrator/AppData/Local/youdao/dict/Application/8.9.3.0/resultui/html/index.html#/javascript:;) [heart](C:/Users/Administrator/AppData/Local/youdao/dict/Application/8.9.3.0/resultui/html/index.html#/javascript:;) [disease](C:/Users/Administrator/AppData/Local/youdao/dict/Application/8.9.3.0/resultui/html/index.html#/javascript:;), n (%) | 117 (8.1) | 14 (7.3) | 0.78 |
| Hypertension, n (%) | 934 (64.8) | 120 (62.5) | 0.57 |
| Diabetes, n (%) | 224 (15.5) | 24 (12.5) | 0.34 |
| Smoking, n (%) | 433 (30.0) | 69 (35.9) | 0.10 |
| History of stroke/TIA , n (%) | 167 (11.6) | 22 (11.5) | >0.99 |
| Prior antiplatelet usage, n (%) | 206 (14.3) | 21 (11.0) | 0.27 |
| SBP, median (IQR), mmHg | 155 (141-169) | 154 (138-167) | 0.26 |
| DBP, median (IQR), mmHg | 85 (77-93) | 86 (77-94) | 0.66 |
| Baseline NIHSS, median (IQR) | 6 (3-12) | 6 (3-11) | 0.93 |
| Prestroke mRS <2, n (%) | 1331 (92.3) | 172 (89.6) | 0.20 |
| Thrombectomy, n (%) | 114 (7.9) | 14 (7.3) | 0.89 |
| DNT ≤ 60 min, n (%) | 1132 (78.5) | 151 (78.6) | 0.64 |

Value are mean ± SD, median (interquartile range), or No. (%) as appropriate.

Abbreviations: mRS, modified Rankin Scale; NIHSS, National Institutes of Health Stroke Scale; TIA, Transient Ischemic Attack; SBP, systolic blood pressure; DBP, diastolic blood pressure; DNT, door-to-needle time.

**Table C** Door-to-needle time, Functional Outcome and Complication Among patients who received IVT during the intervention period between PEITEM and Control Group for Sensitivity Analysis.

| **Variables** | **PEITEM group,**  **No. of Events /**  **Total Patients (%)** | **Control group,**  **No. Of Events /**  **Total Patients (%)** | **Odds Ratio**  **(95% CI)** | **Mean Difference (95% CI)** | ***P* value** |
| --- | --- | --- | --- | --- | --- |
| **Primary outcome** |  |  |  |  |  |
| DNT ≤ 60 min, n (%)^*^ | 888/1093 (81.2) | 500/692 (72.3) | 1.81 (1.18, 2.78) | NA | 0.007 |
| **Secondary outcome** |  |  |  |  |  |
| sICH, n (%)^†^ | 23/1056 (1.0) | 10/649 (1.5) | 1.20 (0.48, 3.01) | NA | 0.697 |
| Favorable functional outcome at 90 days, n (%)^†^ | 538/968 (55.6) | 296/591 (50.1) | 1.39 (1.04, 1.85) | NA | 0.027 |
| Death at discharge, n (%)^†^ | 26/1072 (2.4) | 17/655 (2.6) | 0.85 (0.29, 2.48) | NA | 0.763 |
| mRS at discharge, median (IQR)^†^ | 1 (0-4) | 1 (0-4) | NA | -0.04 (-0.38, 0.30) | 0.817 |
| DNT, median (IQR), min^*^ | 43 (33-57) | 50 (39-63) | NA | -9.05 (-14.65, -3.45) | 0.002 |
| ONT, median (IQR), min^*^ | 155 (110-209) | 163 (112-212) | NA | -2.35 (-13.91, 9.21) | 0.690 |

Value are median (interquartile range), or No. (%) as appropriate.

Abbreviations: CI, Confidence Interval; DNT, door-to-needle time; sICH, symptomatic intracranial hemorrhage; mRS, modified Rankin Scale; ONT, onset-to-needle time.

^*^ Adjusted for patient characteristics (including age, history of stroke/TIA, hypertension, diabetes, atrial fibrillation, coronary heart disease, prior antiplatelet usage, thrombectomy, smoking, prestroke mRS score, and NIHSS score at admission) and hospital characteristics (including hospital grade [tertiary and secondary], stroke unit, teaching hospital status, and annual stroke discharge).

^†^ Adjusted for age, female, history of stroke/TIA, prior antiplatelet usage, thrombectomy, smoking, prestroke mRS score, and NIHSS score at admission.
